# TRPV1 deletion in male mice alters cardiomyocyte ultrastructure without affecting baseline cardiac function

**DOI:** 10.1038/s41598-025-28521-5

**Published:** 2025-12-21

**Authors:** Nolwenn Tessier, Lucille Païta, Christophe Chouabe, Hélène Thibault, Margaux Melka, Mallory Ducrozet, Ribal Al-Mawla, Rania Harisseh, Christelle Léon, Lionel Augeul, Sylvie Dupré-Aucouturier, Gabriel Bidaux, Michel Ovize, Fabien Van Coppenolle, Sylvie Ducreux

**Affiliations:** 1 CarMeN Laboratory- IRIS Team, University Claude Bernard Lyon1, INSERM, INRAE, Bron, 69500 France; 2 Cardiac Intensive Care Unit, Hospices Civils de Lyon, Bron, F-69500 France; 3 Present Address: Grenoble Institut Neurosciences, University Grenoble Alpes, INSERM, U1216, CHU Grenoble Alpes, Grenoble, 38000 France; 4 Present Address: Nantes Université, CHU Nantes, CNRS, INSERM, l’institut du thorax, Nantes, France

**Keywords:** TRPV1, Calcium handling, Heart ultrastructure, Echography, Bibliometric analysis, Nucleus., Cardiology, Cell biology, Physiology

## Abstract

The Transient Receptor Potential Vanilloid 1 (TRPV1) channel is implicated in various cardiovascular processes, including nociception, inflammation, and ischemia-reperfusion injury, yet its role in maintaining baseline cardiac structure and function remains unclear. To address this, we performed a bibliometric analysis of 331 publications (2004–2025) and conducted *in viv*o and ex vivo cardiac phenotyping of sedentary male TRPV1 knockout (TRPV1^⁻/⁻^) and wild-type (TRPV1^⁺/⁺^) mice (8–16 weeks). Echocardiography, patch-clamp electrophysiology, Ca²⁺ handling assays, mitochondrial function tests, and ultrastructural analyses were employed. Bibliometric mapping identified three major research clusters related to TRPV1 in cardiovascular science: ischemia–reperfusion injury, vascular/metabolic regulation, and autonomic control, with no prior studies assessing baseline cardiac function in TRPV1^−/−^ mice. Functional assessments revealed no significant differences between genotypes in echocardiographic parameters, action potential properties, L-type Ca²⁺ currents, Na⁺–Ca²⁺ exchange, or mitochondrial performance. Ca²⁺ transient kinetics exhibited minor alterations without functional impact. Ultrastructural evaluation revealed subtle changes, including slightly longer sarcomeres and altered nuclear morphology (reduced circularity and solidity), while reticulum-mitochondria interfaces remained intact. These findings indicate that deleting TRPV1 does not substantially impair basic cardiac function in young male mice, suggesting a limited role in normal physiology and potential relevance primarily under pathological or stress-induced conditions.

**Supplementary Information:**

The online version contains supplementary material available at 10.1038/s41598-025-28521-5.

## Introduction

TRPV1 (Transient Receptor Potential Vanilloid 1) belongs to the large family of TRP channels (Transient Receptor Potential)^1^. This non-selective cation channel, permeable to calcium ions (Ca^2+^), exerts pleiotropic physiological roles, such as being involved in nociception and baroreflex^2^. Structurally, TRPV1 is a homotetramer composed of six transmembrane domains, with the ion-conducting pore situated between the fifth and sixth segments^2,3^. Functionally, this channel serves as a molecular integrator for various stimuli, including capsaicin (the component responsible for the pungency of hot chilli peppers), resiniferatoxin (RTX; a highly potent analogue derived from *Euphorbia resinifera*), noxious heat (approximately 42 °C), acidosis (pH < 5.9)^2,4,5^. Endogenously, TRPV1 is also regulated by at least phosphorylation, glycosylation^6^, numerous lipids^7^, and calmodulin^8–10^.

While TRPV1 is primarily known for its role in pain sensation, it is also expressed in many non-neuronal tissues (e.g., lung, pancreas, kidney, skeletal muscle)^11^. In the cardiovascular system, studies have reported the presence of TRPV1 in vascular endothelial cells^12^, smooth muscle cells^12^, and C- and Aδ-afferents within the epicardium^13–16^. In cardiac cells, TRPV1 exhibits diverse subcellular localization patterns. In primary neonatal rat cardiomyocytes, TRPV1 localizes to mitochondria^17^. In rat cardiomyoblasts (H9c2 cells), TRPV1 has been detected in the endoplasmic reticulum (ER), particularly at mitochondria-associated endoplasmic reticulum membranes (MAMs), where it may facilitate Ca^2+^ exchange between the ER and mitochondria^18^. In adult mouse ventricular cardiomyocytes, TRPV1 channels were found to be coexpressed with TRPA1 (Transient receptor potential ankyrin type 1) at z-discs, costameres, and intercalated discs^19^. In human iPSC-derived cardiomyocytes, TRPV1 is expressed at the plasma membrane and undergoes internalization in response to inflammatory stimulation, indicating dynamic trafficking in response to stress^20^. Conversely, Hoebart et al. concluded that mouse cardiomyocytes have no functional TRPV1 channel (nor TRPA1)^21^.

Although it remains controversial whether TRPV1 is expressed in adult cardiac muscle tissue per se^22^, the role of TRPV1 in cardiac function has been illustrated on numerous occasions. To our knowledge, several bibliometric analyses related to TRPV1 have been published recently^23–26^. However, none of these have explicitly explored the role of TRPV1 in cardiovascular function to date. To fill this gap, we conducted a bibliometric analysis that maps scientific publications concerning the TRPV1 channel and this research topic from its inception to 2025. Our analysis summarizes and illustrates the temporal features and current knowledge of the related research. Furthermore, it highlights that a specific gap persists, particularly in characterising a TRPV1-deficient mouse heart, which has never been thoroughly investigated under non-pathological conditions.

This observation raises questions regarding the extent to which TRPV1 channels contribute to cardiac function and whether their absence affects the responses of the heart to physiological stimuli. To address these questions, we comprehensively evaluated cardiac function in TRPV1 knockout mice (TRPV1^−/−^) compared to wild-type controls (TRPV1^+/+^). Importantly, TRPV1 knockout animals lack functional TRPV1 channels throughout the entire organism—not only in the heart—allowing us to investigate the global role of TRPV1 in cardiac physiology within a systemic context. Our study included echocardiographic assessments to measure the structural and functional parameters of the heart, as well as electrophysiological recordings from isolated cardiomyocytes to examine the electrical properties reflecting contraction capacity. We also conducted analyses of Ca^2+^ handling and mitochondrial function, given the role of TRPV1 in Ca^2+^ signaling and its potential impact on cellular energetics^27^. Additionally, ultrastructural measurements were carried out to investigate possible changes in cardiac tissue architecture, as TRPV1 has been implicated in cardiac remodeling processes^27^. Thus, the present study aimed to elucidate the role of TRPV1 channels in maintaining normal cardiac physiology. Gaining a deeper understanding of the cardiac functions of TRPV1 channels may offer valuable insights into their broader physiological significance and potential implications in cardiovascular diseases.

## Results

**Fig. 1:**
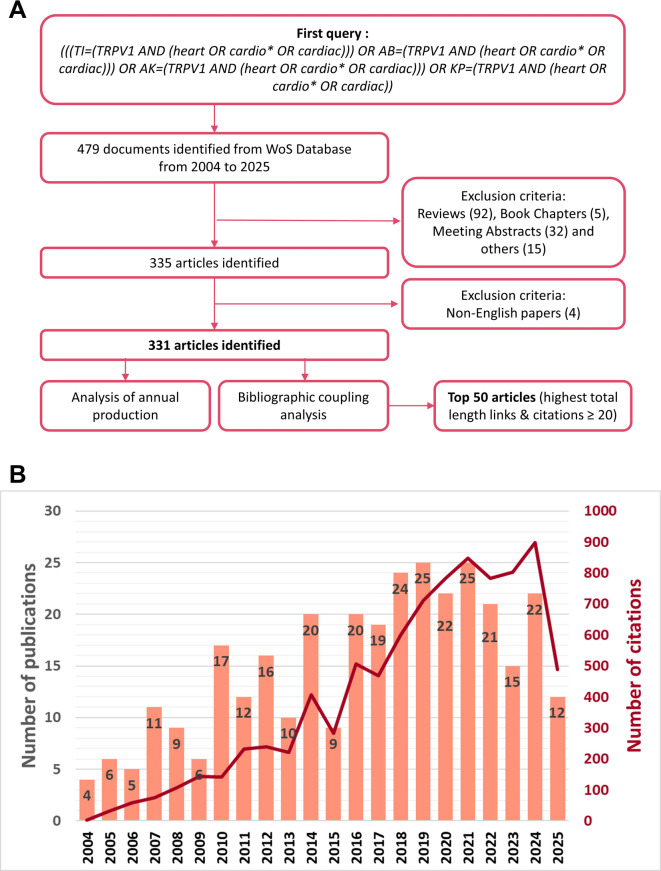
Bibliometric analysis. (**A**) Flowchart showing the literature screening. (**B**) Annual number of publications and citations in TRPV1 and cardiovascular research from 2004 to 2025.

### Bibliometric analysis on the role of TRPV1 in cardiovascular health

We first sought an overview of the evolving research landscape concerning TRPV1 and its role in the cardiovascular system by performing a comprehensive bibliometric analysis. From 2004 to 2025, a total of 331 papers were retrieved from our initial query according to our exclusion criteria, as depicted in Fig. 1A. Initially, we focused on measuring the annual number of publications and related citations (Fig. 1B). By analyzing these metrics, we observed that scientific activity increased from 2004 to 2022, peaking in 2022 after David Julius’s Nobel Prize in 2021. Since then, it seems that scientific production will slow down.

**Fig. 2:**
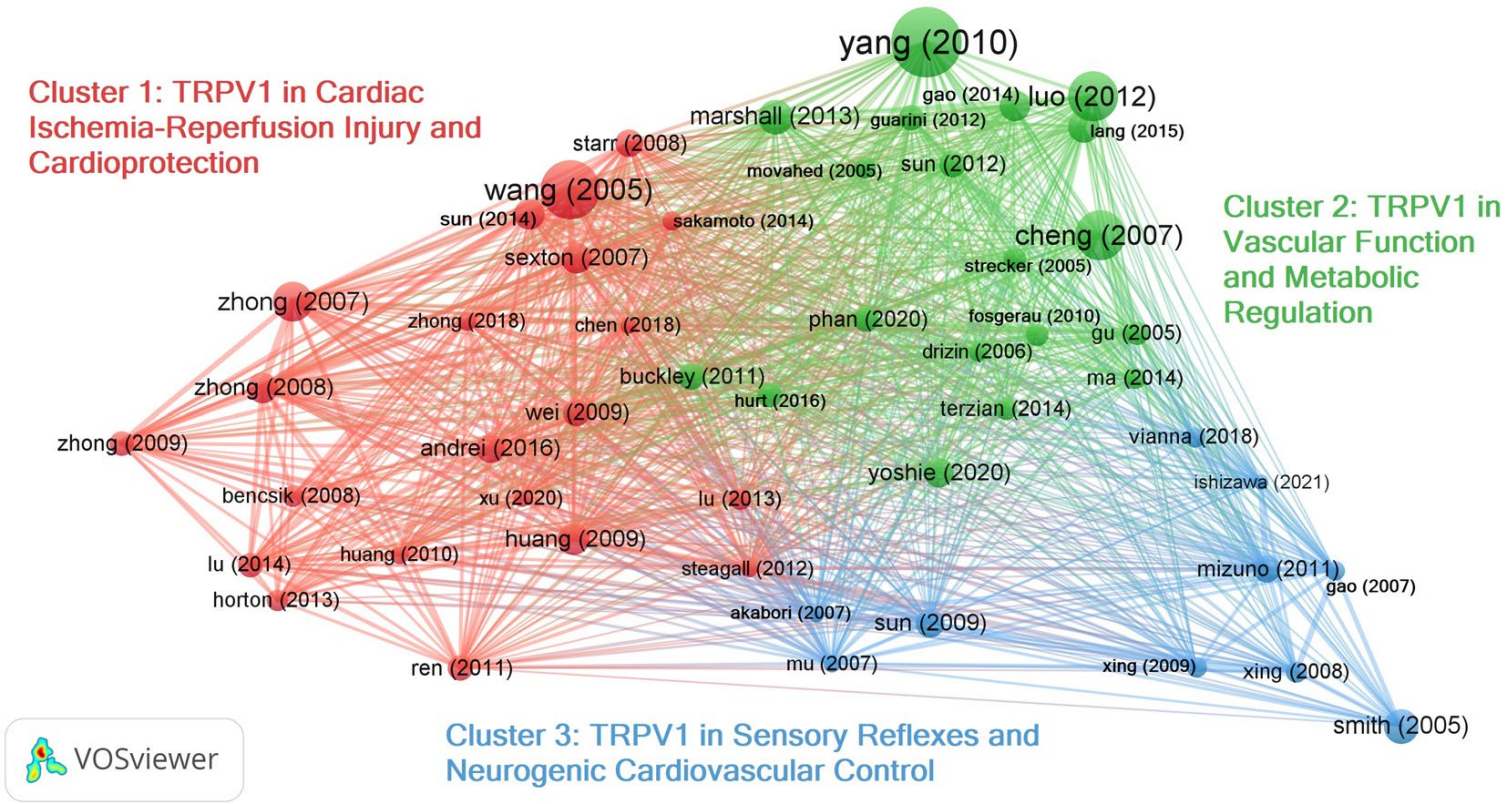
Bibliographic coupling network [2004–2025]. Cluster 1 (red): TRPV1 in ischemia-reperfusion injury and cardioprotection, highlighting its dual role in neuropeptide-mediated protection and calcium-driven injury. Cluster 2 (blue): TRPV1 in vascular and metabolic regulation, linking dietary capsaicin, nitric oxide signaling, and mitochondrial protection to blood pressure control, while also noting context-dependent pathological effects. Cluster 3 (green): TRPV1 in sensory reflexes and autonomic control, emphasizing its role in the exercise pressor reflex, baroreflex, and disease-related sensitization.

Subsequently, we conducted a bibliographic coupling analysis to identify the main clusters of research that have emerged over the past 20 years. In Fig. 2, we identified three main clusters in the literature corpus obtained through our query, which included the Top 50 articles with the highest total link strength and at least 20 citations.

#### Cluster 1: TRPV1 in cardiac ischemia-reperfusion injury and cardioprotection

Twenty-one articles explore the complex role of TRPV1 in cardioprotection, particularly in ischemia-reperfusion injury (I/R). TRPV1 deletion impairs post-ischemic recovery and abolishes preconditioning benefits by reducing neuropeptide release, notably substance P (SP) and calcitonin gene-related peptide (CGRP)^28,29^. TRPV1^−/−^ mice develop larger infarcts, more inflammation, and adverse remodeling after myocardial infarction (MI)^30^. Conversely, TRPV1 activation in cardiomyocytes paradoxically can worsen apoptosis via calcium overload and mitochondrial dysfunction^31^.

Endogenous TRPV1 agonists, like N-oleoyldopamine (OLDA) and 12(S)-HpETE, have been shown to promote cardioprotection by stimulating neuropeptide release^32,33^. TRPV1 also interacts with protease-activated receptors (PAR2) and PKC/PKA pathways to enhance recovery post-injury^34^. In diabetic models, TRPV1 expression is downregulated, leading to impaired postconditioning and recovery, although exogenous CGRP or SP can restore protection^35,36^. Additional findings link TRPV1 to myocardial relaxation through S-nitrosylation of SERCA2a^37^, cold stress-induced hypertrophy^38^, and potential benefits of TRPV1 antagonism in heart failure^39^. TRPV1 further modulates post-MI fibrosis through TGF-β/Smad2 signaling^40^, while spinal TRPV1 activation promotes SP release during ischemia^41^. Morphine preconditioning mitigates injury by suppressing TRPV1 upregulation in dorsal root ganglia^42^, and TRPV1 activation by 20-HETE protects against cardiac dysfunction in sepsis^43^.

#### Cluster 2: TRPV1 in vascular function and metabolic regulation

The second cluster, consisting of 19 articles, focuses on TRPV1’s role in vascular function, blood pressure regulation, and metabolic processes. Dietary capsaicin consistently enhances vascular health by promoting calcium-dependent PKA and eNOS phosphorylation, increasing nitric oxide bioavailability, and facilitating vasorelaxation, ultimately reducing blood pressure^44^. It also mitigates high-salt diet–induced hypertrophy and fibrosis via mitochondrial protection through sirtuin 3^45^ and the upregulation of PPAR-δ^46^. Beyond cardiovascular effects, TRPV1 activation stimulates PGC-1α, boosting oxidative metabolism and exercise endurance, thereby counteracting high-fat diet–induced metabolic dysfunction^47^. Yet the same receptor can also drive pathology: persistent afferent activity post-MI amplifies sympathoexcitation and electrical instability, predisposing to arrhythmias and fibrosis^48^, and TRPV1 deficiency appears protective against pressure-overload hypertrophy^49^. In vascular smooth muscle, TRPV1 may induce constriction and elevate systemic pressure independently of sensory nerves^50^, while its loss in diabetes disrupts coronary blood flow–metabolism coupling through impaired nitric oxide and BK channel signaling^51^. TRPV1 also influences mitochondrial membrane potential and reperfusion injury, with peptide inhibition (V1-cal) reducing infarct size^17^. Pharmacological insights highlight therapeutic potential: endogenous vanilloids such as anandamide modulate vascular tone via sensory nerve TRPV1^52^, and proton-sensitive activation during cardiac acidosis triggers CGRP release, linking ischemic acidosis to vasodilatory neuropeptide signalling^53^. Nicotinic acid, widely used for dyslipidemia, directly activates TRPV1, explaining flushing and revealing clinically relevant vascular effects^54^. Additionally, dihydrocapsaicin induces sustained mild hypothermia suitable for post–cardiac arrest care^55^.

#### Cluster 3: TRPV1 in sensory reflexes and neurogenic cardiovascular control

The third cluster (10 articles) investigates TRPV1’s role in autonomic cardiovascular regulation, particularly through sensory reflex pathways. TRPV1 is highly expressed on group IV skeletal muscle afferents and baroreceptor endings, where it contributes to the exercise pressor reflex (EPR), baroreflex, and related autonomic responses. Altered TRPV1 function has been linked to exaggerated cardiovascular responses in conditions such as heart failure^56^, peripheral arterial occlusive disease^57,58^, hypertension^59^, and type 2 diabetes^60^. Mechanistic studies reveal that TRPV1 mediates afferent activation via metabolic by-products, mechanical stimuli, and modulators such as bradykinin, with intracellular Ca²⁺ signaling and nerve growth factor (NGF) driving sensitization^59,61,62^. Pharmacological interventions—including TRPV1 antagonists such as capsazepine and topical capsaicin—can blunt exaggerated EPR and metaboreflex responses, suggesting therapeutic potential^59,63^. TRPV1 on baroreceptor nerve endings also acts as a mechanosensor for blood pressure changes, and its ablation impairs baroreflex sensitivity^64^. Beyond chronic disease, TRPV1 modulation influences acute outcomes: for example, capsaicin improves survival in hemorrhagic shock by optimizing catecholaminergic responses^65^.

In summary, our bibliographic analysis underscores the dual nature of TRPV1 in cardiovascular health, acting as both a protective and detrimental context-dependent integrator of sensory, vascular, metabolic, and neural inputs. Of the 331 articles analyzed, approximately one-fourth (93 articles) relied on mouse models. Strikingly, none investigated the baseline cardiac function of TRPV1 knockout mice—a reference model available for nearly two decades. This critical gap motivated us to comprehensively characterize the cardiac phenotype of TRPV1^−/−^ mice.

### TRPV1-deficient hearts and cardiomyocytes are fully functional

Given the extensive evidence from Cluster 1 indicating TRPV1’s involvement in ischemia-reperfusion injury and post-MI remodeling, we first sought to assess whether TRPV1^⁻/⁻^ hearts exhibit any signs of remodeling or dysfunction under resting conditions. Firstly, we performed echocardiographic experiments on both TRPV1^⁺/⁺^ and TRPV1^−/−^ mice. As shown in Table 1, no differences were observed between groups at baseline in terms of body mass or heart rate. Echocardiographic measurements, restricted to M-mode parameters, revealed no significant changes in left ventricular end-systolic diameter (LVESD), anterior wall thickness (AWT), posterior wall thickness (PWT), fractional shortening (FS), or left ventricular mass, implying no evidence of cardiac remodeling or systolic dysfunction. Additionally, left ventricular end-diastolic diameter (LVEDD), which provides a dimensional index rather than a direct measure of diastolic function, suggested no changes in chamber size or diastolic filling.

**Table 1:** Echocardiographic analysis of TRPV1^+/+^ and TRPV1^−/−^ mice.

	TRPV1^+/+^	TRPV1^−/−^
Body mass (g)	25.70 [25; 26.4]	23.45 [22.55;27.75]
Heart rate (bpm)	619.0 [601;668]	633.0 [595;676.3]
LVEDD (mm)	3.01 [2.9;3.06]	2.950 [2.843;3.313]
LVESD (mm)	1.19 [1.01;1.55]	1.475 [1.338;1.63]
AWT (mm)	0.8 [0.78;0.88]	0.815 [0.7225; 0.9625]
PWT (mm)	0.88 [0.75;0.93]	0.85 [0.795; 0.915]
FS (%)	59.00 [49;65]	51.00 [47.25;54.50]
LV mass (mg)	62.92 [52.25; 72.75]	61.97 [55.03; 79.13]

Data are presented as median [25th percentile; 75th percentile] and were obtained from 7 TRPV1^+/+^ and 6 TRPV1^−/−^ mice. *LVEDD* left ventricle end-diastolic diameter, *LVESD* left ventricle end-systolic diameter, *AWT* anterior wall thickness, *PWT* posterior wall thickness, *FS* fractional shortening, *LV mass* left ventricle mass. Statistical comparison by Wilcoxon-Mann–Whitney non-parametric tests.

**Fig. 3:**
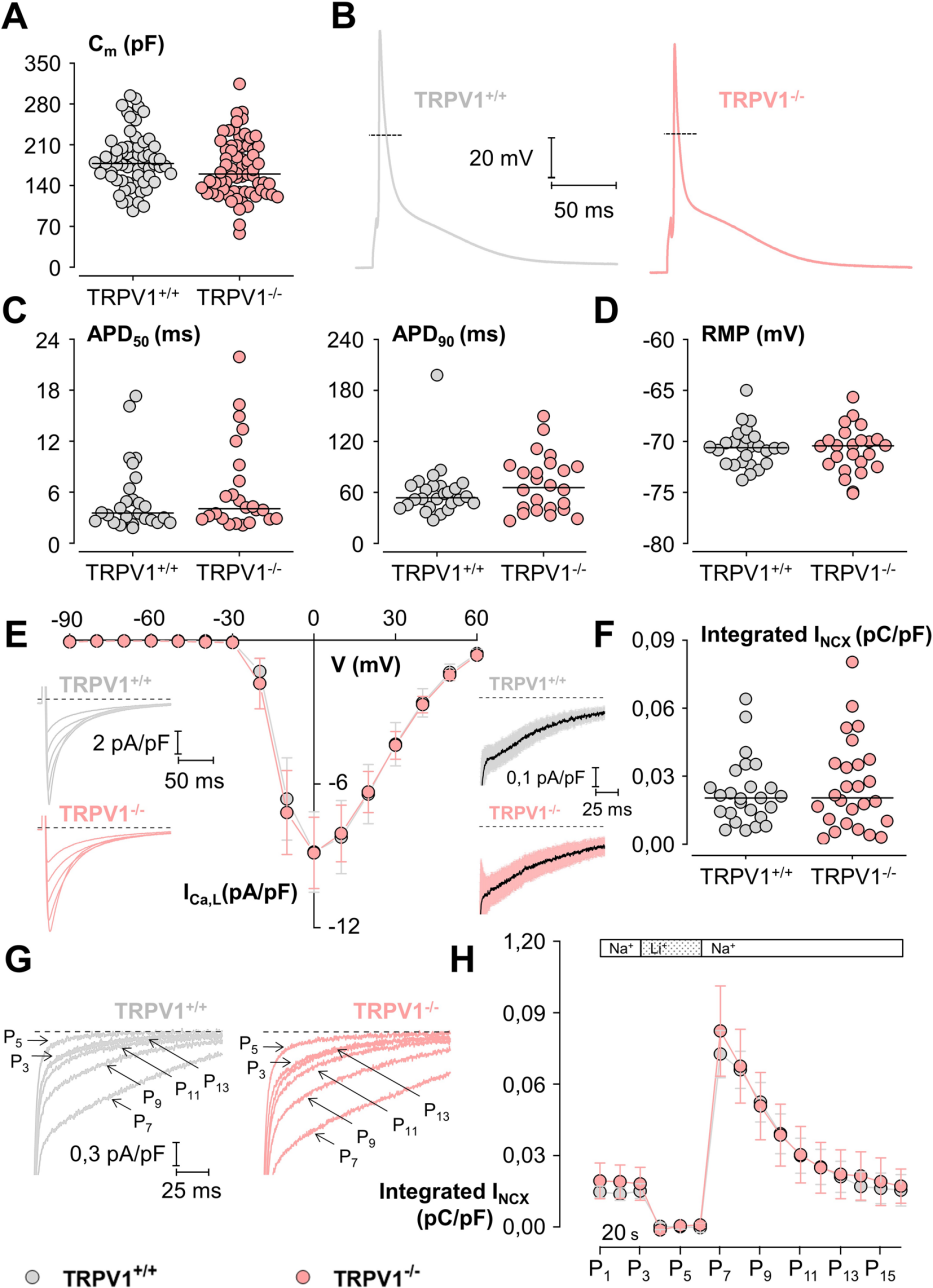
Electrophysiological comparison of TRPV1^+/+^ and TRPV1^−/−^ isolated adult mouse cardiomyocytes. (**A**) Membrane capacitances (C_m_) of TRPV1^+/+^ (*n* = 67 cells from 14 mice; grey) and TRPV1^−/−^ (*n* = 68 cells from 9 mice; pink) cardiomyocytes. (**B**) Representative action potential (AP) of TRPV1^+/+^ and TRPV1^−/−^ cardiomyocytes. (**C**) Action potential durations measured at 50 and 90% of the repolarization phase (APD_50_ and APD_90_) and (**D**) resting membrane potential (RMP) of TRPV1^+/+^ (*n* = 26 cells from 7 mice) and TRPV1^−/−^ (*n* = 24 cells from 4 mice) cardiomyocytes. (**E**) Current-voltage relationships of the peak of L-type Ca^2+^ current (I_Ca, L_) normalized to membrane capacitance from TRPV1^+/+^ (*n* = 16 cells from 3 animals) and TRPV1^−/−^ (*n* = 18 cells from 2 mice) cardiomyocytes. Inserted on the left, representative traces of I_Ca, L_ from TRPV1^+/+^ and TRPV1^−/−^ cardiomyocytes during depolarizing steps spaced 10 mV apart and varying between 0 to + 40 mV (uppermost trace) from a holding potential of −80 mV. (**F**) Sodium-calcium exchange currents (I_NCX_) measured as the integrated form (135 ms integration starting 15 ms after the onset of repolarization) of the Li^+^-sensitive slow tail inward currents recorded when polarizing cells to −80 mV after it has been depolarized 20 ms to −50 mV (to inactivate the fast sodium current), and then 30 ms to + 10 mV (to activate I_Ca, L_) and normalized to membrane capacitance of TRPV1^+/+^ (*n* = 25 cells from 4 mice) and TRPV1^−/−^ (*n* = 26 cells from 3 mice) cardiomyocytes. Inserted on the left, mean traces (± CI 95%) of I_NCX_ from the TRPV1^+/+^ and TRPV1^−/−^ cardiomyocytes, whose integrations are shown on the right. (**G**) Representative traces of inward tail currents normalized to membrane capacitance recorded from TRPV1^+/+^ and TRPV1^−/−^ cardiomyocytes in Na^+^ (P_3_) and Li^+^ (P_5_) external solution and after return in the Na^+^ external solution (P_7_, P_9_, P_11,_ and P_13_). (**H**) Time course of integrated NCX density currents from TRPV1^+/+^ (*n* = 12 cells from 4 mice) and TRPV1^−/−^ (*n* = 13 cells from 4 mice) cardiomyocytes displayed in Na^+^ (P_1_ to P_3_) and Li^+^ (P_4_ to P_6_) external solution and after return in the Na^+^ external solution (from P_7_ to P_16_). Data are expressed as dot plots with the middle line indicating the median (**A**, **C**, **D**, **F**) or as mean ± CI 95% (E, H). Dash lines on panels **E**, **F**, and **G** indicate zero current. Statistical comparison using Wilcoxon-Mann–Whitney non-parametric tests (**A**–**H**).

While echocardiographic data offer a general overview of cardiac function, electrophysiological studies provide crucial insights into the cellular mechanisms underlying heart function. We performed electrophysiological studies on TRPV1^+/+^ and TRPV1^−/−^ isolated adult cardiomyocytes (Fig. 3). We characterized the morphological shape of the cardiomyocytes by measuring the cell capacitance. Median values for membrane capacitances (C_m_) were 177.7 pF and 159.8 pF in TRPV1^+/+^ and TRPV1^−/−^ cardiomyocytes (Fig. 3A). Figure 3B illustrates the action potentials (AP) typically obtained from cardiomyocytes of TRPV1^+/+^ and TRPV1^−/−^ mice in the whole cell patch-clamp configuration. No significant difference was found in the AP average durations at 50% and 90% repolarisation (Fig. 3C), nor in the resting membrane potentials (Fig. 3D) between the TRPV1^+/+^ and TRPV1^−/−^ cells. Alternatively, the application of RTX (10 µM) did not affect the resting membrane potential either in TRPV1^+/+^ (Δ = 0.655 mV) or in TRPV1^−/−^ cells (Δ = 0.45 mV), confirming the absence of TRPV1 channels at the sarcolemma.

Although action potential experiments yield essential data on electrical activity and excitability of cardiomyocytes, the measurement of L-type calcium currents allows for a deeper exploration of the ionic mechanisms underlying cellular depolarization and contraction. We recorded inward L-type Ca^2+^ current (I_Ca, L_) in cardiomyocytes from TRPV1^+/+^ and TRPV1^−/−^ cells (Fig. 3E, left panel) during depolarising steps spaced 10mV apart and varying between 0 and + 40 mV from a holding potential of −80 mV. Figure 3E (right panel) shows the current-voltage relationships of normalised peak I_Ca, L_ to membrane capacitance from TRPV1^+/+^ and TRPV1^−/−^cardiomyocytes. No significant difference in the density of I_Ca, L_ was observed between the two groups of cardiomyocytes, nor in the density of the Na^+^-Ca^2+^ exchange current measured as the lithium-sensitive slow tail current (Fig. 3F). In the following protocol (Fig. 3G,H), NCX currents, which reflect cytosolic Ca^2+^ levels, were utilized to investigate the competition between sarcolemmal Ca^2+^ efflux (via NCX) and SR/ER Ca^2+^ uptake (through SERCA2) during relaxation. Cardiomyocytes experienced Ca^2+^ overload caused by Ca^2+^-induced Ca^2+^ release (CICR) after the lithium blockade of NCX. Initially, each pacing protocol (Fig. 3G, P1–P3) led to a brief cell contraction; however, with the introduction of external Li^+^, they progressively lost their normal contraction ability (Fig. 3G, P4–P6) due to elevated cytosolic Ca^2+^ from inhibited NCX efflux. Upon returning to external Na^+^, the slow tail inward current recorded during repolarization (Fig. 3G: P7 vs. P3) and the integrated NCX current measured after the transition back to external Na^+^ (Fig. 3H) were consistent across the two cardiac genotypes, suggesting that cytosolic Ca^2+^ clearance properties are comparable. Collectively, these findings indicate that the absence of TRPV1 in cardiomyocytes does not affect the contraction capacity of cardiomyocytes.

**Fig. 4:**
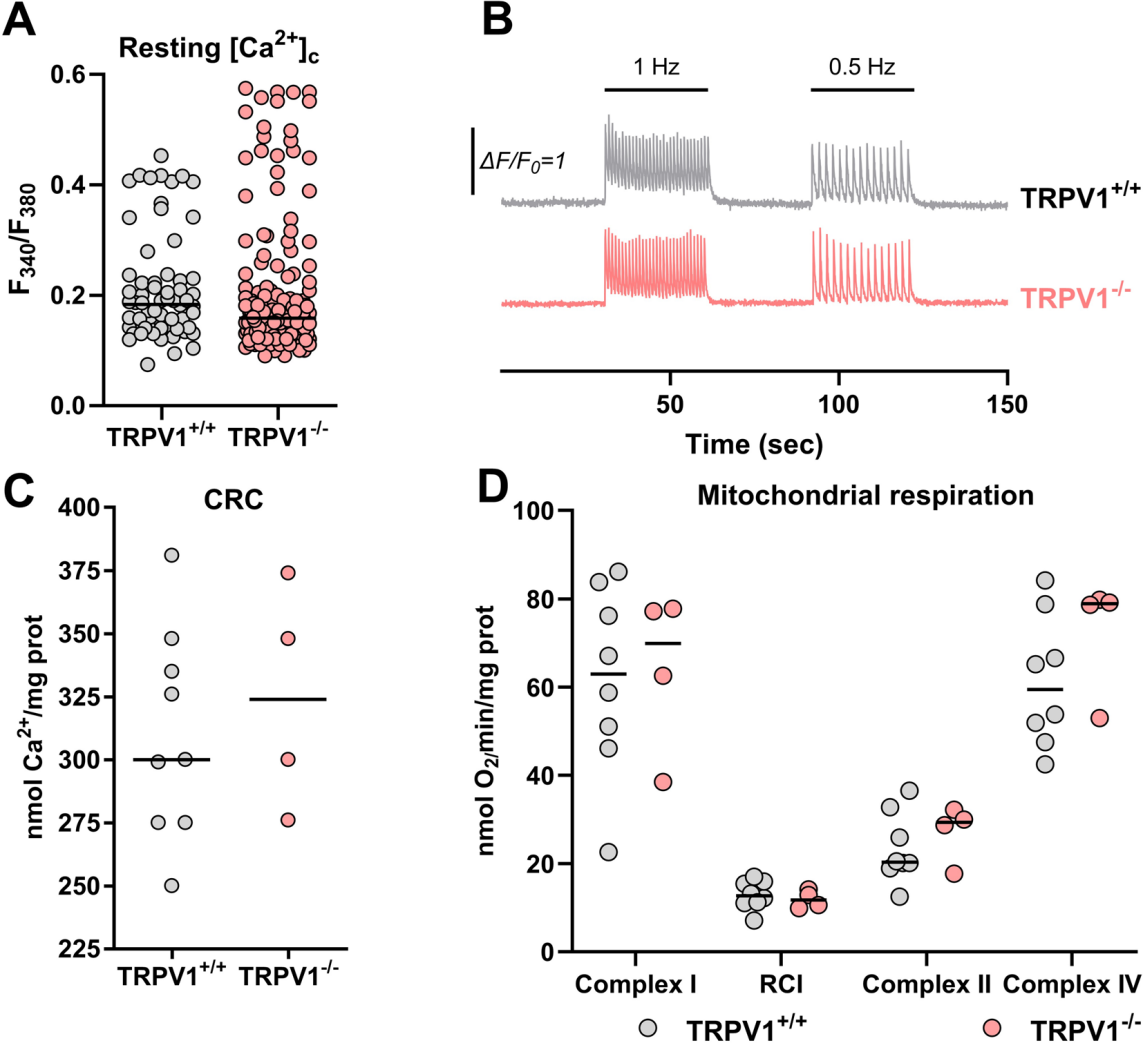
Cytosolic Ca^2+^ handling and mitochondrial function in TRPV1^+/+^ and TRPV1^−/−^ cardiomyocytes. (**A**) Steady-state cytosolic Ca^2+^ concentration ([Ca^2+^]_c)_ expressed as the Fura-2 AM fluorescent ratio for TRPV1^+/+^ (*n* = 66 cells from 3 mice; grey) and TRPV1^−/−^ (*n* = 176 cells from 6 mice; pink) cardiomyocytes. (**B**) Typical curves of [Ca^2+^]_c_ F/F_0_ ratios over time measured with Fluo-4 AM in TRPV1^+/+^ and TRPV1^−/−^ cardiomyocytes in 2 mM Ca^2+^ electro-stimulation buffer for 30 s, followed by 30 s electro-stimulation at 0.50 Hz and 40 V, followed by 30 s resting time, and followed by 30 s electro-stimulation at 1 Hz and 40 V. (**C**) Calcium retention capacity (CRC) expressed as the mean rates from triplicate measurements of Ca^2+^ concentration in nmol Ca^2+^/mg of protein at which opening of the mPTP in TRPV1^+/+^ (*n* = 9 mice) and TRPV1^−/−^ (*n* = 4 mice) mitochondria. (**D**) Mean changes from triplicate measurements in oxygen consumption in nmol O_2_/mg of protein of complex I-linked state 3 respiration, respiratory control index (RCI) (state 3/state 4), complex II-linked state 3 respiration, and complex IV in TRPV1^+/+^ (*n* = 9 mice) and TRPV1^−/−^ (*n* = 4 mice) mitochondria. Data are represented as dot plots, with the middle line indicating the median. Statistical comparison by Wilcoxon-Mann–Whitney non-parametric tests (**A**, **C**, **D**).

Then, we evaluated the cytosolic Ca^2+^ contents in TRPV1^+/+^ and TRPV1^−/−^ adult mouse cardiomyocytes using Fura-2 loading. Figure 4A shows no difference between the resting Ca^2+^ cytosolic concentration of the two strains (0.187 for TRPV1^+/+^ versus 0.159 for TRPV1^−/−^). The following experiments evaluated paced cardiomyocytes, as pacing more closely mimics the natural physiological conditions of the heart. We registered Ca^2+^ cytoplasmic transient using cardiomyocytes paced at 0.5–1 Hz (Fig. 4B). As illustrated in Table 2, we saw no significant difference in the diastolic and systolic efflux, the peak amplitude, and the half-time. In contrast, the time to peak was prolonged, and the time from peak to basal, at the two frequencies, was shortened in TRPV1^−/−^ cardiomyocytes.

**Table 2:** Cytoplasmic Ca^2+^ transient parameters of TRPV1^+/+^ and TRPV1^−/−^ adult mouse cardiomyocytes paced at 0.5–1 Hz.

	0.5 Hz		1 Hz	
	TRPV1^+/+^	TRPV1^−/−^	TRPV1^+/+^	TRPV1^−/−^
F_D_	1.045 [1.006;1.088]	1.121 [1.028;1.021]	1.168 [1.067;1.277]	1.121 [1.028;1.198]
ΔF/F0	0,8179 [0,5927; 1,022]	0,8098 [0,4681;0.8693]	0.6778 [0.4599; 0.9801]	0.7142 [0.4801;0.8693]
AUC	0,4254 [0,3032; 0,5161]	0,3633 [0,2474; 0,4483]	0.2311 [0.1507;0.3005]	0.2304 [0.1656;0.2776]
TTP (s)	0,1962 [0.1039; 0,2698]	0,3667 [0,2536; 0,4796]****	0.1172 [0.1039;0.1462]	0.1386 [0.1167;0.197]**
t_1/2_ (s)	0,3898 [0,3326; 0,4619]	0,3449 [0,3111; 0,3972]	0.2867 [0.2527;0.3111]	0.2826 [0.2509;0.3238]
τ (s)	1,829 [1,759; 1,948]	1,673 [1,562; 1,761]****	0.9016 [0.8751;0.9095]	0.8734 [0.8219;0.9059]*

Data are presented as median [25th percentile; 75th percentile]. *F*_*D*_ diastolic efflux, *ΔF/F0* peak amplitude, *AUC* area under the curve (systolic efflux), *TTP* time to peak, *t*_*1/2*_ half-time, *τ* Time to basal. Statistical comparison by Wilcoxon-Mann–Whitney non-parametric tests: ****p < 0,0001; **p < 0,01; *p < 0,05. At 0.5 Hz, n = 40 TRPV1^+/+^ and 28 TRPV1^−/−^ cells from 4 mice; at 1 Hz, n = 58 TRPV1^+/+^ and 37 TRPV1^−/−^ cells from 5 mice.

Considering the proposed mitochondrial localization of TRPV1^17,66^ and its role in cardioprotection and metabolic regulation, as shown in Clusters 1 and 2, we explored whether deleting TRPV1 impacts mitochondrial function in cardiomyocytes. To assess their functional integrity, we measured calcium retention capacity (which refers to their ability to uptake and store calcium to prevent mitochondrial overload; Fig. 4C) and oxidative phosphorylation (Fig. 4D), both of which were comparable between TRPV1^+/+^ and TRPV1^−/−^ isolated cardiomyocytes.

### TRPV1 elimination affects the nuclear architecture of cardiomyocytes

**Fig. 5:**
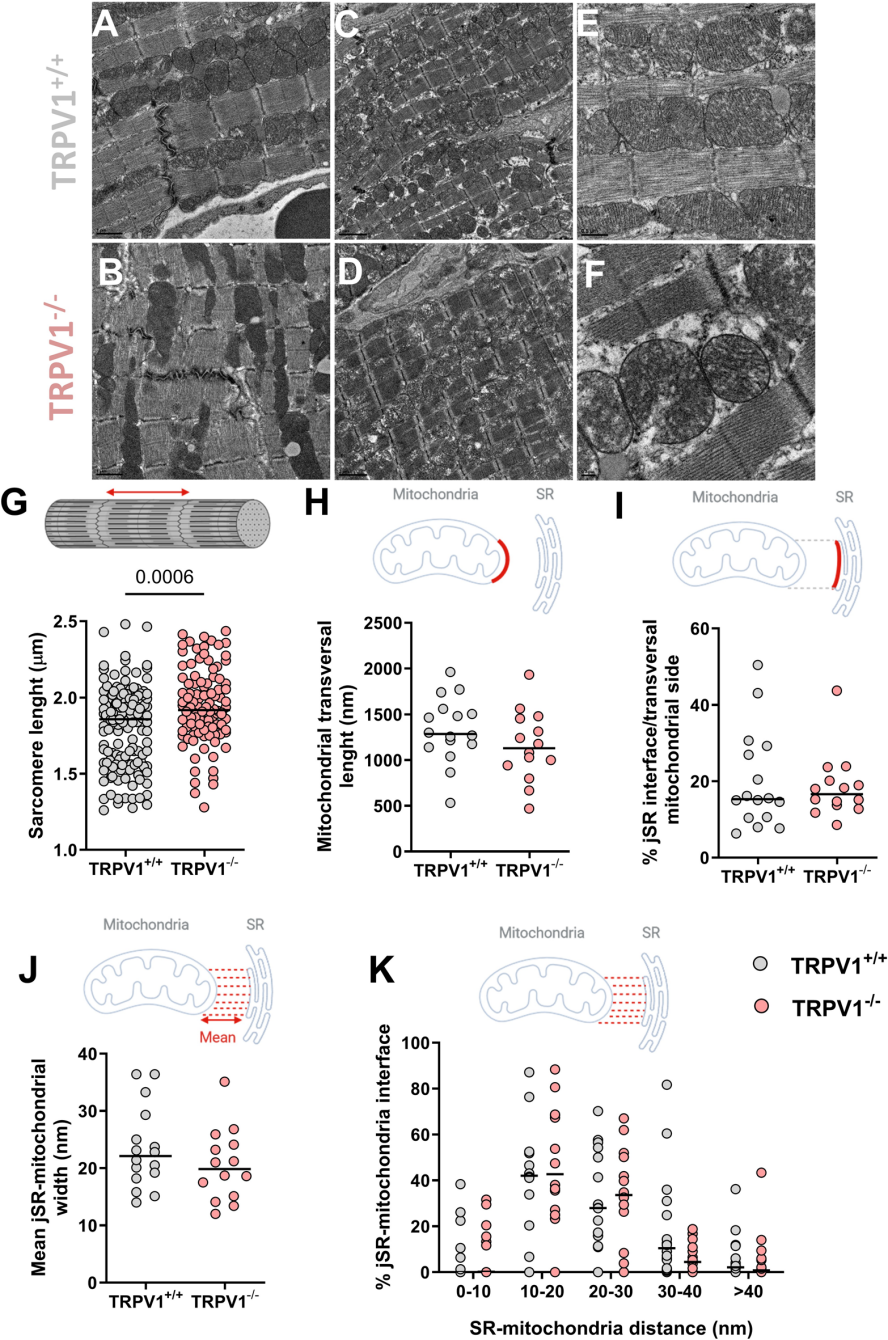
Ultrastructural analysis of cardiac muscle from TRPV1^+/+^ and TRPV1^−/−^ mice. (**A**–**F**) Electron micrographs of myofibrils in cardiomyocytes from TRPV1^+/+^ (**A**–**C**) and TRPV1^−/−^ (**D−F**) mice; scale bars: 1 μm (**A**, **B**), 2 μm (**C**, **D**), 0.5 μm (**E**, **F**). (**G**) Sarcomere length in TRPV1^+/+^ (*n* = 148 images from 7 mice; grey) and TRPV1^−/−^ cardiac muscle (*n* = 102 images from 7 mice). (**H**–**K**) Ultrastructural analysis by electron microscopy of reticulum–mitochondria interactions in TRPV1^+/+^ (*n* = 16 images from 7 mice) and TRPV1^−/−^ cardiac muscle (*n* = 14 images from 5 mice). (**H**) Mitochondrial transversal length. (**I**) Quantification of the jSR–mitochondria interface. (**J**) Mean of the jSR–mitochondria interaction width. (**K**) Frequency distribution of jSR–mitochondria interactions. Data are represented as dot plots, with the middle line indicating the median. Statistical comparison by Wilcoxon-Mann–Whitney non-parametric tests (**G**−**J**) and multiple Mann-Whitney using the Holm-Šídák method (**K**).

**Fig. 6:**
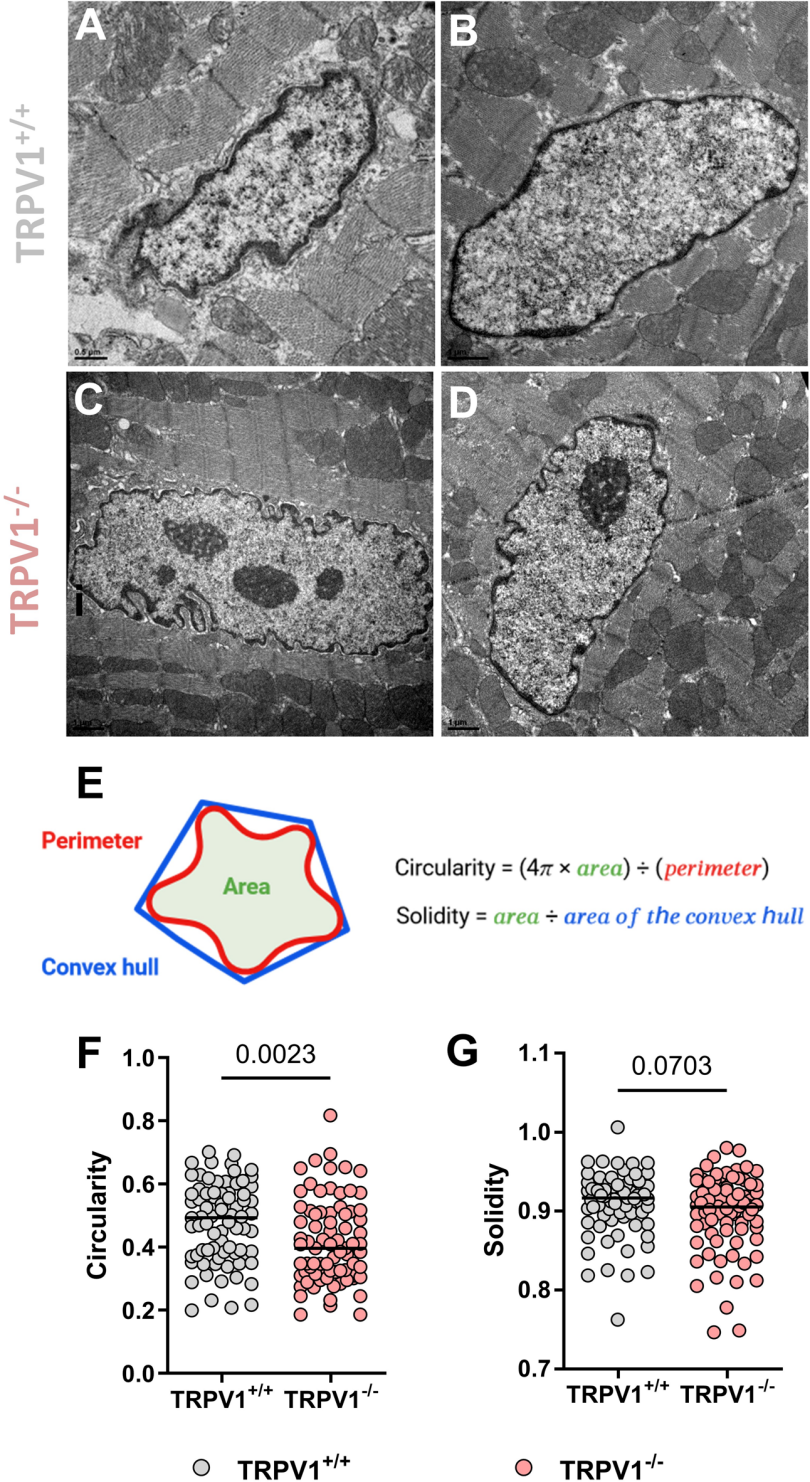
Nuclear shape characteristics from TRPV1^+/+^ and TRPV1^−/−^ cardiomyocytes. (**A**−**D**) Electron micrographs of nuclei in cardiomyocytes from TRPV1^+/+^ (**A**,** B**) and TRPV1^−/−^ (**C**,** D**) mice; scale bar: 1 μm. (**E**) Schematic drawing and formula of nuclear morphometrics. Circularity (**F**) and solidity (**G**) of nuclei from nuclei of TRPV1^+/+^ (*n* = 76 images from 7 mice; pink) and TRPV1^−/−^ (*n* = 79 images from 7 mice; grey) cardiomyocytes. Data are represented as dot plots, with the middle line indicating the median. Statistical comparison by Wilcoxon-Mann–Whitney non-parametric tests (**F**, **G**).

In the last paragraph, we were interested in the ultrastructure of the cardiac tissue (Figs. 5 and 6), in view of the TRPV1’s proposed role in structural remodeling and mechanotransduction pathways, as discussed in Clusters 1 and 3. Sarcomere length in TRPV1^−/−^ mice was found to be around 3% longer than in TRPV1^+/+^ (1.919[1.788;2.094] vs. 1.859[1.585;1.987]; Fig. 5G). We then apprehended the structural interactions between the SR/ER and mitochondria in microdomains called MAMs from electron micrographs of TRPV1^+/+^ and TRPV1^−/−^ hearts. The length of the mitochondrial transversal side (Fig. 5H), the percentage of the jSR–mitochondria interface (Fig. 5I), the mean jSR–mitochondria width (Fig. 5J), and the comparison of the occurrence of interactions (within a given gap width, ranging from 0 to 100 nm; Fig. 5K) were similar in both groups. Since nuclear morphology is tightly linked to cytoskeletal organization and mechanotransduction pathways^67^—which may be influenced by TRPV1-mediated signalling—we finally explored the nuclear shape morphology of cardiomyocytes from the two mouse strains (Fig. 6A−D). Circularity and solidity were used as indicators of structural irregularities (Fig. 6E). The TRPV1 depletion significantly led to fewer circular nuclei (0.3964[0.3103;0.5105] vs. 0.4932[0.3793;0.5704]; Fig. 6F), which also tend to have a reduced solidity (0.9052[0.8750;0.9320] vs. 0.9165[0.8949;0.9324], *p* = 0.0703; Fig. 6G).

## Discussion

This study investigated the role of TRPV1 in cardiac structure and function *via* two complementary approaches: a comprehensive bibliometric analysis and a detailed cardiac phenotyping of TRPV1-deficient mice (TRPV1^−/−^). Our findings indicate that TRPV1 deletion does not significantly impair baseline cardiac performance at either the whole-heart or cellular level. However, it induces subtle but notable ultrastructural changes in cardiomyocytes, particularly affecting nuclear morphology.

Our bibliometric analysis of 331 papers published between 2004 and 2025 reveals a growing interest in the role of TRPV1 in cardiovascular health. Three distinct research clusters have emerged: the role of TRPV1 in cardioprotection during ischemia-reperfusion injury, the involvement of TRP channels in vascular and metabolic regulation, and the contribution of TRPV1 in sensory reflexes and autonomic control. Depending on the context, these studies highlight TRPV1 as a biphasic modulator of cardiac health—beneficial when activated through sensory neuropeptide signaling, but potentially harmful when improperly stimulated in cardiomyocytes or during pathological stress, emphasizing the need for further research to fully understand the mechanisms behind these contrasting effects. Despite extensive use of mouse models, none have deeply examined the baseline cardiac function of TRPV1 knockout mice—a gap that motivated this work.

Our electrophysiological and Ca^2+^ handling experiments on isolated cardiomyocytes revealed no significant differences in action potential characteristics, L-type calcium current density, or resting cytosolic Ca^2+^ concentration between TRPV1^+/+^ and TRPV1^−/−^ cardiomyocytes. However, there was a slight increase in the time to peak and a decrease in the time from peak to basal in TRPV1^−/−^ cells. These minor variations suggest that while TRPV1 channels may influence the kinetics of Ca^2+^ transients, they do not substantially impact overall Ca^2+^ handling in cardiomyocytes. For comparison, TRPV4^−/−^ mouse ventricular myocytes have been shown to have a reduced action potential duration compared to wild-type mice^68^. This indicates that TRPV1 channels may not be essential for fundamental cardiomyocyte electrophysiology or Ca^2+^ homeostasis under basal conditions.

While we observed no functional deficits in TRPV1^−/−^ hearts or cardiomyocytes, our ultrastructural analysis revealed significant alterations in sarcomere length and nuclear morphology. More specifically, TRPV1^−/−^ cardiomyocytes showed a slight increase in sarcomere length — probably too minor (3%) to be physiologically relevant, at least at rest. However, reduced circularity and solidity nuclear indices may reflect altered chromatin organization or mechanotransduction^67,69^. Interestingly, these nuclear abnormalities resemble those seen in lamin A/C-related disorders, which are associated with cardiomyopathies such as dilated cardiomyopathy and conduction system disease^70–71^. In cardiac tissue, nuclear envelope integrity plays a key role in sensing mechanical stress and maintaining genomic stability^72,74^. Disruption of nuclear structure has been linked to impaired contractility, arrhythmogenesis, and increased vulnerability to damage, as observed in dilated cardiomyopathy^75^. Therefore, even in the absence of obvious dysfunction, the nuclear changes we observed in TRPV1^-/-^ hearts may represent latent structural vulnerabilities that could become relevant under stress or pathological conditions. On the other hand, the reticulum-mitochondria interfaces remained intact in TRPV1^−/−^ mice. This indicates that TRPV1 channels are not essential for maintaining these key structural features, even though they can modulate them^18^.

It is important to note that TRPV1$^{ -/- }$ mice lack TRPV1 expression systemically—not just in the heart. TRPV1 is expressed in sensory neurons, vascular smooth muscle, endothelial cells, and various visceral organs, where it modulates neurogenic inflammation, autonomic tone, and metabolic responses, as highlighted in our bibliometric Clusters 2 and 3. Thus, the absence of cardiac dysfunction in TRPV1$^{ -/- }$ mice may reflect compensatory mechanisms involving other ion channels or signaling pathways that help preserve normal heart function under physiological conditions. For instance, TRPV2 and TRPV1 share an 86.2% homology, with minor differences in the pore loop region and various similarities; i.e., both channels are activated by high temperature (52 °C and 42 °C, respectively)^76^. Several papers have reported a severe decline in cardiac function and an impairment of Ca^2+^ handling in TRPV2-deficient mice compared with controls^77,78^. In the present work, we show that eliminating TRPV1 does not lead to a critical alteration in cardiac function. We can speculate that, because of their different expression levels in the heart (TRPV2 > TRPV1^72^, TRPV1 participates in a finer Ca^2+^ tuning than TRPV2, making both channel types complementary targets for a better understanding of the cellular processes occurring during myocardial dysfunction. This functional redundancy could explain the preserved echocardiographic parameters in TRPV1$^{ -/- }$ mice. Besides, our bibliometric Cluster 1 points to autonomic plasticity and alternative neuropeptide pathways, such as those involving CGRP and substance P, which may further buffer the loss of TRPV1-mediated regulation. It is also worth considering that ketamine, used for mouse sedation, even at sub-anesthetic doses (e.g., 80 mg/kg IP), may modulate sympathetic tone and mask subtle genotype-dependent differences. Moreover, our echocardiographic assessment was limited to M-mode parameters, which primarily reflect systolic geometry and function. While LVEDD provides a dimensional index of diastolic filling, it does not provide a comprehensive evaluation of diastolic performance. Future studies using Doppler-based imaging would be valuable for a more complete assessment of cardiac function.

One noteworthy limitation of our study is the use of only young, sedentary male mice (8–16 weeks). TRPV1 signaling may vary significantly with age, sex, and physiological context^79,80^. Preliminary data from older TRPV1$^{ -/- }$ mice (32–36 weeks) showed no significant differences in L-type Ca^2+^ currents (see Supplementary Fig. S1), suggesting that this parameter is age-independent. However, sex-specific differences in TRPV1 function remain largely unexplored in cardiac tissue. For example, TRPV1 expression is higher in female bladder arterioles and increases with age, suggesting hormonal and developmental regulation^81^. In skeletal muscle, TRPV1$^{ -/- }$ mice exhibit sex-dependent phenotypes, with exercise negatively affecting slow-twitch muscles in females^82^. Recent single-nucleus transcriptomic profiling of adult mouse hearts has also revealed sex-specific molecular signaling and identified a novel cardiomyocyte population associated with CCNA2-mediated cardiac regeneration^83^. This suggests that TRPV1 may interact with sex-dependent regenerative pathways, which could be masked in male-only cohorts. Finally, the scarcity of female data in most TRPV1-related mouse cardiac studies (only 3 of 93 studies in our bibliographic dataset included females) represents a significant gap in the literature. These insights highlight the need for future studies to include both sexes and account for hormonal status to fully elucidate TRPV1’s role in cardiac physiology and disease susceptibility.

## Conclusion

In summary, TRPV1 deletion in young male mice does not impair baseline cardiac function but changes cardiomyocyte ultrastructure, especially nuclear shape. Compensatory mechanisms likely counterbalance these effects, and TRPV1 may only assume pathological or regulatory roles under stress conditions, in older animals, or in female groups, warranting further investigation into its therapeutic potential.

## Methods

### Bibliometric analysis

#### Scope and aim of the analysis

We aimed to determine the research structure and evolution of TRPV1 in the cardiovascular context. The bibliometric analysis was designed following the guidelines of Donthu et al.^84^ and the methodology of Chalet et al.^85^, as previously described^86^.

#### Data collection

Clarivate Web of Science© was used to collect data (Copyright Clarivate 2024WoS). All data were exported from the WoS database on August 5, 2025.

We applied the following query:

(((TI=(TRPV1 AND (heart OR cardio* OR cardiac))) OR AB=(TRPV1 AND (heart OR cardio* OR cardiac))) OR AK=(TRPV1 AND (heart OR cardio* OR cardiac))) OR KP=(TRPV1 AND (heart OR cardio* OR cardiac)).

The corpus included only Articles in English. Review-type articles and book chapters were also excluded from the analysis to avoid the misclustering of non-related methods co-citations. A total of 331 documents were obtained, ranging from 2004 to 2025, from 178 sources and 2005 authors. Included documents are displayed in Supplementary Table S1.

#### Bibliographic coupling method

WoS viewer was used to display bibliometric networks represented through mapping and clustering to perform the analysis. For the bibliographic coupling, the clusters were explored on the articles with the highest total link strength, corresponding to the link of an item with other items in a network. This coupling was carried out on articles with a minimum of 20 citations. The visualization map was built with the top 50 papers with the highest total link strength. Three clusters were obtained. Included references grouped by clusters are displayed in Supplementary Table S2. The nomination and analysis of the clusters were conducted based on the top 50 selected articles.

### Ethics statement

All the procedures were performed in accordance with the principles and guidelines established by the European Convention for the Protection of Laboratory Animals and were approved by the Lyon 1 Claude Bernard University Committee for Animal Research (BH2012-65; date of approval: December 2012; DR2018-51 - APAFIS#17798; date of approval: November 2018). This study was carried out in accordance with the ARRIVE guidelines.

### Animals

TRPV1^+/+^ and TRPV1^−/−^ male mice (8–16 weeks, 20–30 g) were used throughout the study. C57Bl6J TRPV1^+/+^ mice were used as controls (Charles River, L’Arbresle, France), and TRPV1^−/−^ (B6.129 × 1-Trpv1^tm1Jul^IJ) mice were bred in our laboratory from two couples bought from The Jackson Laboratory. Genotyping of TRPV1^+/+^ and TRPV1^−/−^ mice was realised according to the KAPA Mouse Genotyping Kit (KR0385 – v2.13).

### Isolating adult murine ventricular cardiomyocytes

Adult TRPV1^+/+^ and TRPV1^−/−^ mice were heparinised (50 UI/kg body weight IP, Panpharma) and sacrificed by cervical dislocation without anesthesia. The animal’s death was confirmed by the cessation of heartbeat and the loss of reflexes. A thoracotomy was performed, and the heart was collected. Following the cardiomyocyte isolating protocol of O’Connell et al.^87^, the heart was immediately perfused using the Langerdorff technique with perfusion buffer at 37 °C as previously described^88^. The perfusate was then switched to the enzyme solution. This solution was perfused for 8 min. From the digested heart, the ventricles were excised. Pieces were gently stirred and filtered to collect an isolated cardiomyocyte suspension. After isolation, cardiomyocytes underwent several baths of cell isolation buffer in which Ca^2+^ concentration was gradually increased (40 µM to 900 µM). Then, cardiomyocytes can be plated on plastic dishes (Ibidi, Biovalley) coated with 10 µg/mL laminin (BD Biosciences) or non-coated for 1 h before being used for Ca^2+^ imaging and electrophysiology experiments, respectively.

### Electrophysiology

For electrophysiological recordings of left ventricular cardiomyocytes isolated from TRPV1^+/+^ and TRPV1^−/−^ adult mice, all procedures were performed using the conventional whole-cell patch-clamp technique^89^ and following the methods, including the indirect estimation of the activity of SERCA2, already described in Gonnot et al.^90^.

### Echocardiography

Echocardiography was performed in 8–12-week-old TRPV1^+/+^ and functional TRPV1^−/−^ male mice. Animals were sedated using ketamine (one dose of ketamine 80 mg/kg IP) rather than fully anesthetized as previously described^91^. This approach was chosen to minimize motion artifacts while preserving stable cardiac function. Importantly, we avoided inhalational anesthetics, as TRPV1 channels possess a known binding domain for isoflurane^92^ and are sensitive to halothane^93^. The use of ketamine thus reduced the risk of confounding interactions with TRPV1 signaling during functional assessments. Images were acquired with a 13-MHz linear-array transducer with a digital ultrasound system (Vivid 7, GE Medical Systems). LV end-diastolic diameter (LVEDD), LV end-systolic diameter (LVEDSD), anterior wall thickness (AWT), and posterior wall thickness (PWT) were obtained from M-mode tracings at the level of the papillary muscles. Fractional shortening (FS) was calculated using the formula (LVEDD-LVESD)/LVEDD*100 to assess LV global function. LV mass was calculated using the formula 1.05 × [(AWT + PWT + LVEDD) ^3^ − LVEDD^3^]^94^.

### Ca^2+^ experiments

#### Ca^2+^ measurements

For cytosolic Ca^2+^ imaging, cardiomyocytes were incubated in Ca^2+^-containing buffer (CCB; in mM: 140 NaCl, 5 KCl, 10 HEPES, 1 MgCl_2_, 2 CaCl_2_, 10 glucose; adjusted to pH 7.4) with 1 µM Fura-2 AM at 37 °C. Measurements were performed as previously described^95^.

#### Ca^2+^ Transients

For these experiments, cardiomyocyte preparation and transient recordings were performed as described^95^, except that cardiomyocytes were loaded at 37 °C with Fluo-4 AM (5 µM) for 30 min. Fluo-4 AM was excited at 488 nm by an argon-ion laser. The emitted fluorescent light was collected at a wavelength of 525/50 nm using a highly sensitive GaAsp detector. Images were acquired with a resonant scanner (16 frames per second). Ca^2+^ transient parameters (area under curve, amplitude, time to peak, half decay time, slope, and time to basal) were obtained using a custom-written macro on Matlab software as previously described^96^.

### Calcium retention capacity

The calcium retention capacity (CRC), an index of the resistance of the mPTP to opening following matrix Ca^2+^ accumulation, was determined on freshly isolated adult mouse cardiomyocytes as previously described^97^.

#### Mitochondrial oxidative phosphorylation

Oxygen consumption in freshly isolated adult mouse cardiomyocytes was measured at 25 °C using a Clark-type oxygen electrode (Oroboros oxygraph, Austria). Measurements on cardiomyocytes (250 µg proteins) were carried out using a previously described method^98^.

### Electron microscopy

For the ultrastructural study, isolated hearts were sliced transversely into 1-mm sections. Sample preparation and the analysis of ER-mitochondria interfaces were then performed following the methods already described by Tessier et al.^18^. The sarcomere length was measured from Z-line to Z-line in ImageJ. The nuclear morphometrics were computed with ImageJ, according to a procedure adapted from Harvey et al.^99^. Circularity, which defines how far from a perfect circle a shape is relative to the perimeter, was determined by the equation: Circularity = (4 ×) ÷ (). Solidity, which describes the feature known interchangeably in the literature as nuclear morphology irregularities, was derived from the equation: Solidity = ÷ ℎ ℎ, in which the convex hull is generated by drawing a line between two neighbouring protrusions and measuring the area contained within.

### Statistics

Statistical analysis was performed with GraphPad Prism 10 software. When the normality test was passed, t-tests were performed for comparison between the two groups, while ANOVA followed by Tukey’s multiple comparisons test was used to analyze differences among multiple groups. When the normality test failed or the sample size was too small, Wilcoxon-Mann–Whitney non-parametric tests were used to compare two groups, and the Kruskal–Wallis test followed by Dunn’s multiple comparisons test was used to analyze differences among multiple groups. For experiments involving electrophysiological recordings, calcium imaging, and ultrastructural measurements, comparisons were conducted at the cellular level to reflect intrinsic cell-to-cell variability. For all other experiments, including echocardiographic and biochemical assessments, data were analyzed per animal. The difference was considered significant when the p-value was < 0.05.

## Supplementary Information

Below is the link to the electronic supplementary material.

Supplementary Material 1

Supplementary Material 2

Supplementary Material 3

## Data Availability

The data that support the findings, including statistical analyses and reagents used, are available from the corresponding author upon reasonable request.
