# Correction: TRPV1 deletion in male mice alters cardiomyocyte ultrastructure without affecting baseline cardiac function

**DOI:** 10.1038/s41598-026-37014-y

**Published:** 2026-02-02

**Authors:** Nolwenn Tessier, Lucille Païta, Christophe Chouabe, Hélène Thibault, Margaux Melka, Mallory Ducrozet, Ribal Al-Mawla, Rania Harisseh, Christelle Léon, Lionel Augeul, Sylvie Dupré-Aucouturier, Gabriel Bidaux, Michel Ovize, Fabien Van Coppenolle, Sylvie Ducreux

**Affiliations:** 1 CarMeN Laboratory- IRIS Team, University Claude Bernard Lyon1, INSERM, INRAE, Bron, 69500 France; 2 Cardiac Intensive Care Unit, Hospices Civils de Lyon, Bron, F-69500 France; 3 Present Address: Grenoble Institut Neurosciences, University Grenoble Alpes, INSERM, U1216, CHU Grenoble Alpes, Grenoble, 38000 France; 4 Present Address: Nantes Université, CHU Nantes, CNRS, INSERM, l’institut du thorax, Nantes, France

Correction to: *Scientific Reports* 10.1038/s41598-025-28521-5, published online 21 December 2025

The original version of this Article contained errors in the name of the authors: Nolwenn Tessier Lucille Païta, Christophe Chouabe, Hélène Thibault, Margaux Melka, Mallory Ducrozet, Ribal Al-Mawla, Rania Harisseh, Christelle Léon, Sylvie Dupré-Aucouturier, Gabriel Bidaux, Michel Ovize and Sylvie Ducreux. The names were incorrectly given as Tessier Nolwenn, Païta Lucille, Chouabe Christophe, Thibault Hélène, Melka Margaux, Ducrozet Mallory, Al-Mawla Ribal, Harisseh Rania, Léon Christelle, Dupré-Aucouturier Sylvie, Bidaux Gabriel Ovize and Michel Ducreux Sylvie.

The original Article has been corrected.

