# Supplementary material for: TRPV1 deletion in male mice alters cardiomyocyte ultrastructure without affecting baseline cardiac function
Source: Sci Rep. 2025 Dec 21;15:45781. doi: 10.1038/s41598-025-28521-5 (PMC12756331; doi:10.1038/s41598-025-28521-5)
Supplement: Supplementary file 3 — Supplementary Material 3 [file 41598_2025_28521_MOESM3_ESM.pdf]

TRPV1 Deletion in Male Mice Alters Cardiomyocyte Ultrastructure Without Affecting Baseline Cardiac Function

Nolwenn Tessier, Lucille Païta, Christophe Chouabe, Hélène Thibault, Margaux Melka, Mallory Ducrozet, Ribal Al-Mawla, Rania Harisseh, Christelle Léon, Lionel Augeul, Sylvie Dupré-Aucouturier, Gabriel Bidaux, Michel Ovize, Fabien Van Coppenolle and Sylvie Ducreux

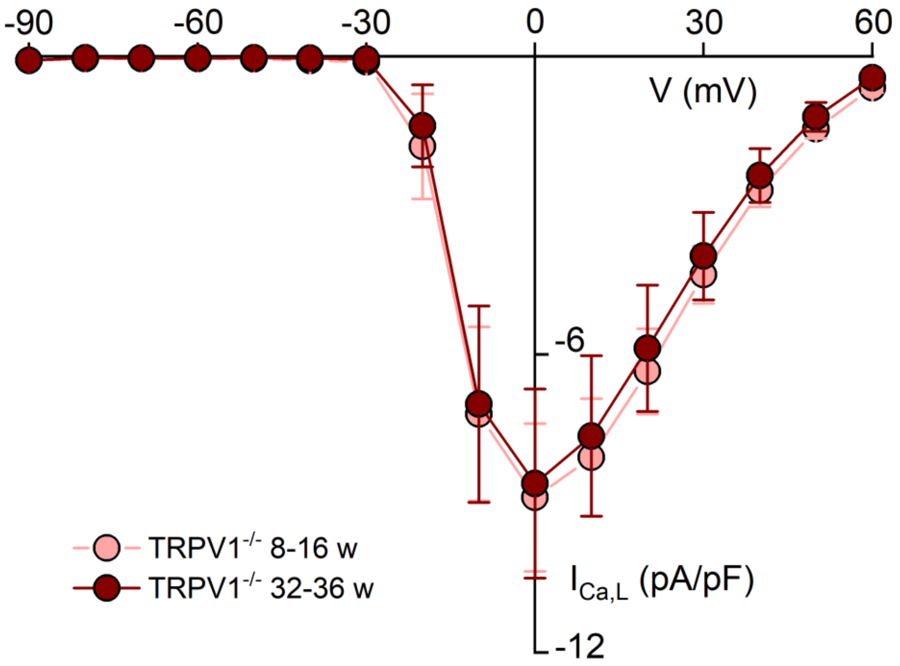

Fig. S1. Electrophysiological comparison of 8–16-week-old and 32–36-week-old TRPV1$^{-/-}$ isolated adult mouse cardiomyocytes. Current-voltage relationships of the peak of L-type Ca^{2+} current ($I_{Ca,L}$) normalized to cardiomyocyte membrane capacitance from 8–16-week-old (n = 18 cells from 2 animals) and 32-36-week-old (n = 10 cells from 2 animals) TRPV1$^{-/-}$ mice.
